# A Novel Technique for Thoracic Endovascular Graft Explantation

**DOI:** 10.1177/15569845231158659

**Published:** 2023-03-05

**Authors:** Asher A. Frydman, Maude Paquet, Chuce Xing, Matthew Valdis, Adam Power, Michael W. A. Chu

**Affiliations:** 1 Division of Cardiac Surgery, Western University, London, ON, Canada; 2 Division of Vascular Surgery, Western University, London, ON, Canada

**Keywords:** aorta, thoracic endovascular aortic repair, graft thrombosis, graft explant, re-sheath

## Abstract

Thoracic endovascular aortic repair (TEVAR) explantation remains a challenge due to endovascular graft ingrowth into the aortic wall with time. Surgical access into the aortic arch can be difficult either via sternotomy or thoracotomy, and proximal barbs become engaged firmly into the aortic wall. Explantation often requires extensive thoracic aortic resection, sometimes from the distal aortic arch to the abdominal aorta, followed by reconstruction, risking injury to surrounding neurovascular structures and even death. In cases of blunt thoracic aortic injury, the original injury is often healed, and failed TEVAR could theoretically be removed when thrombotic complications occur. We present a novel technique to facilitate TEVAR recapture with limited distal thoracic aorta replacement.

## Graphical abstract

**Visual abstract:**
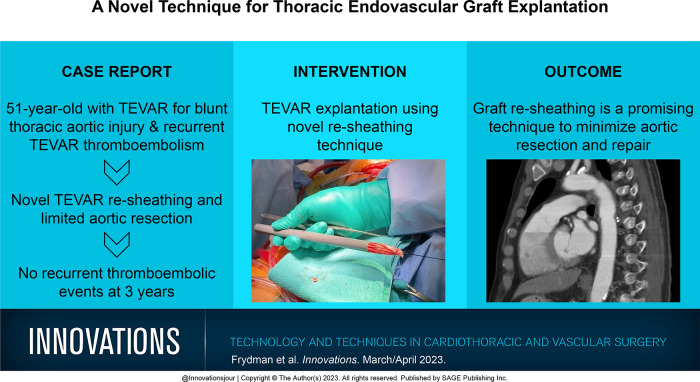

## Introduction

Thoracic endovascular aortic repair (TEVAR) graft explantation can be required for infection or thrombus accumulation resulting in thromboembolic events.^1^ It often requires extensive aortic exposure to remove a graft deeply embedded in surrounding tissues and to have access to the proximal aspect of the graft where the barbs are securely anchored into the aortic wall. This can be challenging, resulting in increased risk of injury to surrounding structures, including the phrenic, recurrent laryngeal, and vagus nerves, as well as risk of paraplegia and death.^1^

## Case Report

**Fig. 1.**
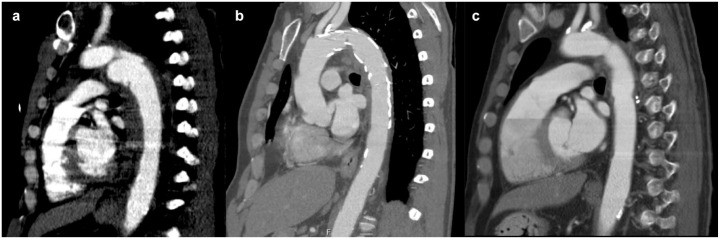
Sagittal computed tomography scans demonstrating (a) grade 3 blunt thoracic aortic injury after motor vehicle collision, (b) thoracic endovascular aortic repair stent with evidence of thrombosis, and (c) postoperative thoracic endovascular aortic repair explanation and aortic reconstruction with intact anastomoses.

**Fig. 2.**
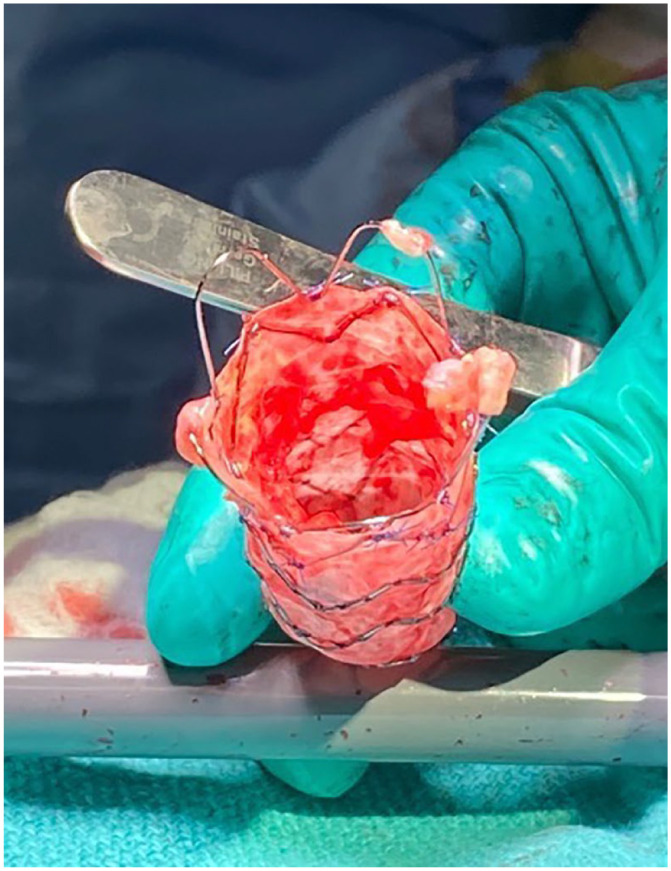
Proximal aspect of explanted 24 × 105 mm Zenith Alpha graft (Cook Medical, Bloomington, IN, USA) priorly implanted in the proximal aorta to distal arch.

A 51-year-old patient was transferred to our emergency department following a motor-vehicle collision. Notable on computed tomography (CT) was the presence of a grade 3 blunt thoracic aortic injury (BTAI; Fig. 1a). He was taken to the operating room emergently for endovascular repair and treatment of his polytrauma. A 24 × 105 mm Zenith Alpha graft (Cook Medical, Bloomington, IN, USA) was deployed just distal to the left subclavian artery, covering the pseudoaneurysm successfully (Fig. 2). A surveillance CT was performed at 3-month follow-up, demonstrating thrombus accumulation in the graft (Fig. 1b). Despite various regimens of systemic anticoagulation and antiplatelet agents, the patient had 2 embolic events within 18 months of TEVAR insertion requiring repeated left iliofemoral artery thromboembolectomy. It was therefore decided to explant the graft.

**Fig. 3.**
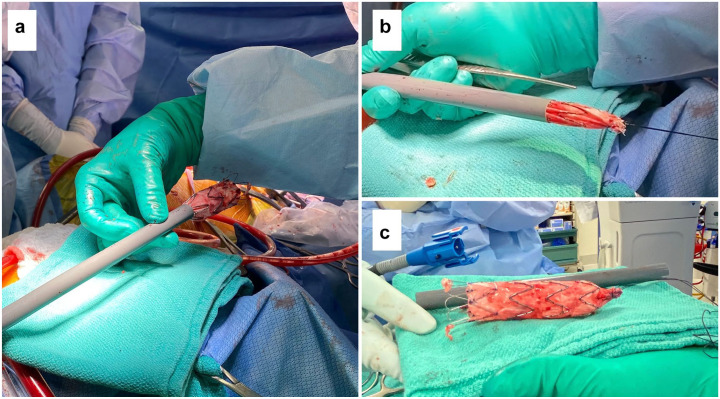
Postoperative demonstration showing (a) 24 × 105 mm Zenith Alpha graft (Cook Medical, Bloomington, IN, USA) being re-sheathed through a GORE dry sheath cover (W. L. Gore & Associates, Inc., Newark, DE, USA), (b) re-sheathed stent graft being pulled through the GORE dry sheath by silk suture to prolapse out the distal end, and (c) successfully explanted stent graft removed from the GORE dry sheath.

He was taken to the operating room 2 years later for TEVAR graft explant and proximal descending aortic reconstruction with a 22 mm Ante-Flo graft (Terumo Aortic, Sunrise, FL, USA). This was done through a left posterolateral thoracotomy through the fourth intercostal space under deep hypothermic circulatory arrest. The distal arch and proximal descending aorta were dissected free from the extensive pulmonary adhesions. Proximally, a portion of the aorta was dissected circumferentially but was quite adherent to the surrounding tissues. The left carotid artery and left femoral vein were used for full cardiopulmonary bypass. Circulatory arrest was achieved when the patient was cooled to 20 °C and antegrade cerebral perfusion via the left carotid artery. Clamps were placed at the base of the carotid artery and descending thoracic aorta. The descending aorta was incised, and the distal part of the stent graft was prolapsed out into the left chest and allowed to flush out thromboembolic debris. A circumferential silk suture was sewn around the distal end of the endostent to collapse it. We then passed the suture through an 18 Fr GORE dry sheath cover (W. L. Gore & Associates, Inc., Newark, DE, USA) and pulled the distal end of the endostent into the sheath cover to collapse, re-sheath, and slide it out of the distal arch and proximal descending aorta with minimal resistance (Fig. 3, Supplemental Video 1, Supplemental Video 2). The distal end of the stent graft was recaptured easily, with the most proximal barbs requiring mild tension and a rotational force to recapture the remaining stent graft successfully. Thrombus was present within the graft. After debriding some fibrinous tissue from within the aorta and irrigating copiously, we reconstructed the distal arch and proximal descending aorta with a short 22 mm Ante-Flo graft in an end-to-end fashion with running polypropylene. After reinitiating proximal flow, rewarming the patient, and repositioning the distal clamp on the Ante-Flo graft, we reconstructed the distal end with running polypropylene suture. Finally, the descending thoracic aorta was covered with a bovine pericardial patch. Hemostasis was achieved, incisions were closed, and the patient was transferred back to the intensive care unit in stable condition on no inotropic support. Cardiopulmonary bypass time was 108 min, and total circulatory arrest time was 39 min with continuous antegrade cerebral perfusion.

The patient’s postoperative course was complicated by respiratory failure requiring prolonged mechanical ventilation and delirium, but he eventually recovered and was discharged home after a month of hospitalization. Repeat echocardiogram showed a normal ejection fraction, normal valvular function, and no wall motion abnormalities. Postoperative CT of the heart revealed the in situ graft with intact anastomoses (Fig. 1c, Supplemental Video 3). At 3-year follow-up, he was doing well with no recurrent distal thromboembolic complications.

## Discussion

The technique we used for stent removal consists of re-sheathing the endostent into a large-bore sheath cover by pulling it in with silk sutures around the distal graft. Re-sheathing allows secure dissection of the graft from surrounding structures with minimal collateral aortic trauma. When the sheath approaches the proximal aspect of the graft, the fixation barbs are captured relatively smoothly inside the sheath. This technique allows collapsing of the stent that is deeply embedded in surrounding structures, without requiring extensive dissection proximally into the aortic arch. This minimizes the need for extensive aortic reconstruction with its associated risks including nerve injury, paraplegia, infection, and bleeding.^1^ This technique could be performed with a variety of large-bore vascular access sheaths or sheath covers. However, the sheath covers must be stiff enough to enable graft recapture. Notably, this technique has several caveats to be aware of. The rate of success is unknown, and in some cases, the barbs or graft might be fully incorporated into surrounding structures and impossible to be removed via re-sheathing. Should significant resistance be encountered when attempting to re-sheath the proximal graft, one may need to proceed with wide excision and arch reconstruction. The re-sheathing technique should not be applied for graft infection because all infected material and surrounding tissue must be removed for source control. Aortic dissection and rupture remain an important risk with this procedure if it is not employed cautiously, since the proximal aspect of the sheath is not visualized when inserted into the aorta. Therefore, the technique should be avoided in patients with previous aortic dissection. Patients with heavy thrombus burden should probably not undergo this technique as well, as the risk of precipitating thromboembolic complications when re-sheathing the graft remains a concern. We believe this risk can be reduced with careful CT examination and thorough irrigation, flushing, and debridement before and after graft explant.

Although endovascular repair of BTAI offers minimally invasive repair that causes less physiologic disturbances in the setting of multisystem trauma, complications exist. TEVAR graft thrombosis can occur secondary to graft oversizing, especially in the 30% range. Young trauma patients have a higher risk due to smaller, more conical, and tightly angulated aorta.^2^ Graft design is also important. In 2017, the Food and Drug Administration recalled the 18 to 22 mm Zenith Alpha Thoracic Endovascular Graft from Cook due to thrombus buildup. The Instruction for Use removed the indication to use this graft for BTAI.^3^ When symptomatic graft thrombosis occurs, the definitive treatment is explantation, although endovascular relining or extra-anatomic bypass can be used as temporizing measures.^4^ When thromboembolic events occur secondary to graft thrombus buildup, explant must be considered if graft relining fails.

Hence, in symptomatic TEVAR thrombosis or thromboembolic events, using the re-sheathing technique for graft explantation can help recapture the proximal barbs and separate the graft from the surrounding tissue. This could be used as an adjunct in graft explant as it can decrease the morbidity associated with extensive aortic dissection proximally into the aortic arch.
